# Rapid development of strong, persistent, spatiotemporally extensive cortical synchrony and underlying oscillations following acute MCA focal ischemia

**DOI:** 10.1038/s41598-020-78179-4

**Published:** 2020-12-08

**Authors:** Ellen G. Wann, Anirudh Wodeyar, Ramesh Srinivasan, Ron D. Frostig

**Affiliations:** 1 Department of Neurobiology and Behavior, University of California, Irvine, CA USA; 2 Center for the Neurobiology of Learning and Memory, University of California, Irvine, CA USA; 3 Department of Cognitive Science, University of California, Irvine, CA USA; 4 Department of Statistics, University of California, Irvine, CA USA; 5 Department of Biomedical Engineering, University of California, Irvine, CA USA

**Keywords:** Neuroscience, Diseases, Neurology

## Abstract

Stroke is a leading cause of death and the leading cause of long-term disability, but its electrophysiological basis is poorly understood. Characterizing acute ischemic neuronal activity dynamics is important for understanding the temporal and spatial development of ischemic pathophysiology and determining neuronal activity signatures of ischemia. Using a 32-microelectrode array spanning the depth of cortex, electrophysiological recordings generated for the first time a continuous spatiotemporal profile of local field potentials (LFP) and multi-unit activity (MUA) before (baseline) and directly after (0–5 h) distal, permanent MCA occlusion (pMCAo) in a rat model. Although evoked activity persisted for hours after pMCAo with minor differences from baseline, spatiotemporal analyses of spontaneous activity revealed that LFP became spatially and temporally synchronized regardless of cortical depth within minutes after pMCAo and extended over large parts of cortex. Such enhanced post-ischemic synchrony was found to be driven by increased bursts of low multi-frequency oscillations and continued throughout the acute ischemic period whereas synchrony measures minimally changed over the same recording period in surgical sham controls. EEG recordings of a similar frequency range have been applied to successfully predict stroke damage and recovery, suggesting clear clinical relevance for our rat model.

## Introduction

Nearly 800,000 strokes occur in the United States each year, many of which result in long-term disability and death. Of these stroke cases, as many as 87% are ischemic (i.e., resulting from an obstruction of blood flow)^1^. A relatively small proportion of ischemic stroke cases receive intravenous tissue plasminogen activator (tPA), the most established neuroprotective intervention^2^. Neuroprotective agents, including tPA and stent-retriever thrombectomy, are most effective when administered early and improve stroke outcome when delivered within 4–6 h after ischemic onset^3,4^. However, reperfusion therapy delivered 6–16 h after ischemic onset has been shown to improve stroke outcome in humans with extensive collateral circulation and slow infarct growth^5^.

Using neuronal activity measures to determine the proportion of acute ischemic cases eligible for neuroprotective and reperfusion interventions and the appropriate utilization of these interventions may be helpful for improving stroke outcomes. Characterizing neuronal function during stroke supplements injury-based diagnostics^6,7^, indicating the importance of identifying distinguishing signatures of post-ischemic dysfunction. Electrophysiological recordings of neuronal activity are capable of describing in detail evoked and spontaneous cortical function and are, therefore, optimal for measuring how cortical function is disrupted after ischemic onset.

Middle cerebral artery (MCA) occlusion, the most common type of human stroke, varies widely and is not appropriately modeled by a single kind of animal experiment^8^. Our permanent MCA occlusion (pMCAo) model, involving a ligation and subsequent severing of the rat’s distal (M1 segment) MCA, results in evoked activity deficits and cortical infarct at 24 h but a smaller infarct than in more common, proximal MCA occlusion models^8,9^. Utilizing more diverse, moderate animal models of ischemia like ours is necessary to mimic specific features (e.g., distal location) of human ischemia accurately and understand stroke presentation differences in the human population.

Stroke damage occurs after a complex sequence of cellular and molecular pathophysiological events that progresses over time and space post ischemic onset^10^, yet very little is known about the spatiotemporal scale of large neuronal network activity within and outside the ischemic region. Therefore, our research aims for the first time to continuously map the development of spatial and temporal neuronal activity patterns in the minutes and hours after ischemic onset using a 32-microelectrode array spanning depths of S1 and neighboring cortical regions within MCA territory. Determining the evolution of spontaneous neuronal activity changes post pMCAo identified acute ischemic signatures of cortical dysfunction even prior to regional evoked activity impairments.

Synchrony, a measure of temporally coordinated neuronal activity across recording locations, is modulated during many cognitive and motor processes, indicating its critical importance for cortical function^11,12^. Our study sought to evaluate spatiotemporal synchrony during the acute period after distal MCA occlusion because of its ability to characterize the spatial and temporal patterning of spontaneous neuronal activity across widely distributed neuronal networks. Determining if and how synchrony is affected by ischemia is critical for understanding the role of temporal coordination in the function of large-scale networks and functional impairments associated with ischemia.

Using a rodent model of pMCAo, we found increased spatially extensive spatiotemporal synchrony within minutes after ischemic onset driven by temporally distinct bursts of low multi-frequency oscillations. Therefore, large-scale spontaneous neuronal synchrony is a rapidly emerging signature of ischemia and may antecede evoked neuronal activity impairments in distal MCA ischemic models (i.e., pMCAo).

## Methods

All experiments were in accordance with NIH guidelines and approved by the University of California, Irvine’s Animal Care and Usage Committee.

### Surgical preparation

Adult (300–400 g) male Sprague Dawley rats (Charles River Laboratories, Wilmington, MA) were anesthetized with sodium pentobarbital [55 mg/kg, body weight (b.w.)] and anesthesia was maintained throughout experimentation by supplemental injections of sodium pentobarbital (14 mg/kg, b.w.) every hour. Dextrose (5%, 3 mL) and atropine (0.05 mg/kg b.w.) were injected every 6 h, and anesthetized body temperature was maintained at 37 °C using a self-regulating thermal blanket.

High spatial resolution functional imaging (intrinsic signal optical imaging, ISOI) was first conducted through a thinned skull preparation (6.5 by 8 mm imaging window of the left somatosensory cortex) in all subjects in order to position electrodes relative to the C2 whisker functional representation of Posterior Medial Barrel Subfields (PMBSF) of somatosensory cortex. Visualization of cortical functional representations by ISOI and guided electrode placement was performed as in previous studies to assure that electrodes were placed in similar locations within MCA territory in all animals (a detailed description can be found in previous studies from the laboratory^13,14^). Since MCA provides blood to somatosensory cortex^10^, the organization of the PMBSF allowed for ischemic conditions to be induced in a region where cortical activity can be reliably evoked and assessed.

Two craniotomies were then performed on each animal: a 1.5 mm by 5 mm craniotomy centered over the C2 whisker functional representation for electrode lowering and a 2 mm by 2 mm craniotomy above the ascending (M1) branch of the MCA for occluding the MCA.

### Surgical occlusion (pMCAo)

MCA ischemia was achieved as previously described in detail^9,15,16^. Prior to electrode insertion (see “Electrophysiology”), a surgical ligature was inserted beneath the M1 segment of the left middle cerebral artery (MCA) in the MCA craniotomy window with minimal dura removal. After baseline recordings, the threads were tied and MCA transected to permanently block the distal MCA. Separate surgical sham control animals underwent all of the aforementioned procedures except for pMCAo.

### Electrophysiology

Multi-site recordings were acquired using a fixed array of 32 electrodes made from insulated 35 μm stainless steel wire (HML and VG bond coating insulated California Fine Wire, Grover Beach, CA) attached to a stainless-steel ground wire. Comparable to an earlier study from the laboratory^17^, electrodes were threaded in groups of four through polyimide guide tubes and adjusted for the electrode tips to be 250, 600, 1200, or 1500 μm from end of the polyimide tubes distanced each 0.65 mm apart via a custom 3D printed mold. Impedance of electrodes was maintained at approximately 150 kΩ. Signals were amplified and digitized at a 22 kHz sample rate (downsampled to 2.2 kHz for analysis) (SnR system, Alpha Omega, Nazareth, Israel). An adjustment period of 1 h occurred after electrode placement before recording. Raw signals were acquired continuously for either a 30 min baseline period followed by 5 h after ischemic onset (pMCAo group) or the same duration with no occlusion (surgical sham group). In separate experiments, raw signal acquisition occurred for a total of 3 s (1 s before, during, and after stimulus onset) per trial for baseline and post pMCAo stimulation trials (refer to “Sensory (whisker) stimulation”). For all recordings, raw signals were band-pass filtered for local field potential (LFP) (1–300 Hz) or multiunit activity (MUA) (300–3000 Hz) using a two-pole Butterworth function in Matlab. MUA was transformed post-hoc into single events by detecting threshold crossings three times the baseline root mean squared (RMS). MUA firing rate was defined as the number of events per second. All noisy channels (8.85% of electrodes) were eliminated from analyses and any 60 Hz noise was removed from analyses using a notch filter function in Matlab.

### Sensory (whisker) stimulation

In separate animals than discussed in spontaneous activity analyses, sensory stimulation was delivered before and after pMCAo. Post pMCAo stimulation ranged intermittently from 0 to 2 h after ischemic onset in one group of rats or from 3 to 5 h after ischemic onset in another group of rats to determine if evoked activity properties changed from ischemic onset to 5 h after pMCAo. Similar to the stimulation delivery protocol from past studies, single whisker (C2) was deflected for 5 pulses (9 degrees) at 5 Hz every 27 s for a total of 100 trials before occlusion and a total^9,16^.

### Experimental design

#### Evoked properties

For each recording location, trials were averaged during baseline and post pMCAo. LFP response properties measured included the absolute value of the negative peak (minimum magnitude), the time to negative peak (latency), and the positive peak value (maximum magnitude). The MUA response properties assessed included the mean evoked firing rate (magnitude) and time to the first 1 ms bin of peak firing rate (peak latency). As measured previously^14^, magnitude of MUA was determined from a 50 ms epoch beginning 7 ms after the stimulus minus the spontaneous firing rate from a 300 ms epoch 350 ms prior to any stimulation. Statistical analysis of LFP and MUA response properties were restricted to electrodes with evoked responses.

#### Spatiotemporal synchrony

In surgical sham animals and animals with no sensory stimulation delivered, neuronal activity synchrony was assessed by cross-correlating the spontaneous LFP of each electrode with all other electrodes in the recording array for a 1 s time window given a 100 ms range of time lags. For a given time sequence (1 s), the maximum cross-correlation coefficient was reported as well as the time lag (up to ± 100 ms) that resulted in the maximum cross-correlation coefficient. Cross-correlations comparing each location with all other recording locations were then repeated for each 1 s of the baseline and the 5 h post pMCAo time series totaling 21,600 correlations for each pair of microelectrodes. To simplify the 32 by 32 matrix generated for each recorded second and quantify how cross-correlations changed over the entire recording time series (refer to “Results”), all locations’ maximum cross-correlation coefficients were averaged for each recorded second and reported as a function of time. The average cross-correlation coefficient measure was then normalized to the z-transformation of the total baseline to compare average cross-correlation coefficient trends across animals.

#### Power analysis and power burst extraction

Complex Morlet wavelet analysis was applied to derive the time-varying, frequency power structure for a logarithmic set of frequencies ranging from 1 to 200 Hz during the LFP time series. To facilitate comparison across animals, power was normalized by the z-transformation with respect to the total baseline^18^.

After baseline normalization, temporally discrete bursts of high power were identified to determine the cortical oscillatory dynamics of acute ischemia. The power threshold set was the RMS of each frequency’s power estimate. A burst was defined as when power exceeded the RMS threshold for 100 or more consecutive ms, and the burst length was defined by the duration of the interval where power was above the RMS power threshold.

#### Spatiotemporal synchrony after power burst extraction

Identification of the time points encompassed in power bursts of each frequency was utilized to scramble each electrode’s LFP at corresponding time points. Scrambling occurred by replacing the LFP time series with randomly generated values within the inter-quartile range (IQR) of the LFP magnitude. Cross-correlations were then rerun on concatenated LFP comprised of non-bursting LFP and scrambled (bursting epochs) LFP.

### Statistical analyses

To analyze evoked activity properties and histological differences, we utilized nonparametric tests because of insufficient evidence of normally distributed data. When appropriate, we applied Kruskal–Wallis and Wilcoxon’s signed-rank tests (Matlab functions kruskalwallis and ranksum respectively).

### Spatiotemporal synchrony

For each subject, 1000 bootstrap samples were generated by sampling (with replacement) from baseline and post-occlusion periods separately^19^. The mean was then found across subjects prior to forming a test statistic of the difference between the baseline and post-occlusion for each resample. The difference between baseline and post-occlusion was considered significant if the bootstrap resample 95% confidence interval (CI) did not encompass the null statistic (no difference between baseline and post-occlusion periods)^20^. As a result of test-statistic used, confidence intervals but no exact p-values are reported for these analyses.

### Histological preparation

Histology was collected 24 h after pMCAo to assess infarct volume at a time point similar to other studies^10^. A fixed and cryoprotected flattened cortex was sectioned transversely for all horizontal electrode locations to be visualized in the same section as the infarcted region. For each slice, infarct volume was estimated by quantifying the area (ImageJ area tool) and extrapolating to the 1.65 mm depth of rat PMBSF^14^. In each animal, an average infarct volume and an infarct percentage (of cortex) was approximated as well as the percentage of infarct volume to cortical volume.

## Results

### Robust MUA and LFP evoked responses during acute post pMCAo period

**Figure 1:**
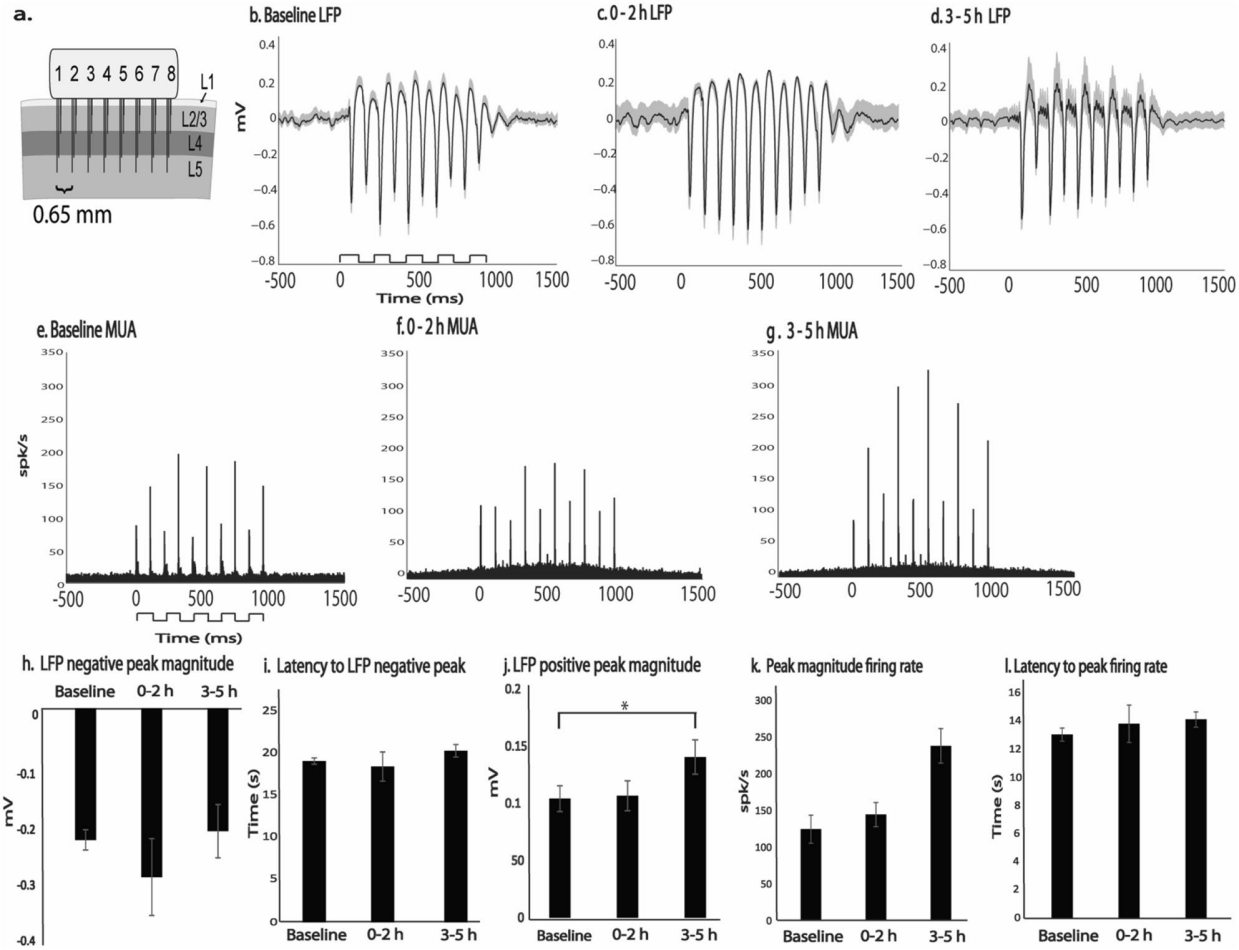
Evoked LFP and MUA responses are robust throughout the acute post pMCAo period. Evoked LFP (**b**–**d** and **h**–**j**) and MUA (**e**–**g** and **k**–**l**) for peak recording location Layer 4 electrodes are similar when stimulation is delivered at baseline and 0–2 h. Multi-site electrode array (**a**) recordings sample 8 horizontal locations across the MCA ischemic cortex each 0.65 mm apart and at depths of 300 μm (layer 2/3), 700 μm (layer 4), 1100 μm (layer 5a), and 1500 μm (layer 5b). Layer 4 evoked LFP and MUA responses are depicted prior to ischemia and either directly after ischemic onset (0–2 h stimulation, *n* = 5) or at a 3 h delay after ischemic onset (3–5 h stimulation, *n* = 6). LFP positive peak magnitude (*p < 0.05) and MUA peak magnitude (*p* = 0.07) was increased compared to baseline when stimulation was delivered 3–5 h after pMCAo. However, 3–5 h evoked response measures of LFP negative peak magnitude, LFP latency to negative peak, and MUA latency to peak firing rate were not significantly different than baseline. Regardless of when sensory stimulation was delivered relative to pMCAo, whisker stimulation was delivered in a 5 pulse 5 Hz train as indicated by the step function in b. The gray outline in (**b**–**d**) demonstrates the standard error trial average evoked LFP responses relative to the mean response in solid black. Peristimulus time histograms in (**e**–**g**) show MUA events detected when the peak of filtered trace surpassed a threshold of 3 times the RMS of the baseline.

To determine the effect of pMCAo induced ischemia on evoked activity in the MCA territory, we intermittently stimulated the C2 whisker prior to ischemia and either directly after ischemic onset (0–2 h stimulation, *n* = 5) or at a 3 h delay after ischemic onset (3–5 h stimulation, *n* = 6). Both MUA and LFP evoked responses continued to be robust throughout the acute post pMCAo period (Fig. 1a–g). In trial averaged traces from peak recording location Layer 4 electrodes, LFP response properties of negative peak magnitude, negative peak latency, and positive peak magnitude were assessed as measures of evoked subthreshold activity. No statistically significant difference was observed between baseline, 0–2 h, and 3–5 h stimulation in either LFP negative peak magnitude (*H* = 0.41,* p* = 0.67, Kruskal–Wallis test; Fig. 1h) or LFP latency to negative peak (*H* = 0.30,* p* = 0.74, Kruskal–Wallis test; Fig. 1i). The magnitude of the LFP positive peak response significantly increased in 3–5 h stimulation as compared to baseline and 0–2 h stimulation trials (*H* = 4.37, *p* = 0.03, Kruskal–Wallis test; Fig. 1j). Similarly, compared to baseline stimulation, MUA peak firing rate increased approaching significance in 3–5 h stimulation (*H* = 3.13, *p* = 0.07, Kruskal–Wallis test; Fig. 1k) but not in 0 to 2 h stimulation (*H* = 0.22, *p* = 0.64, Kruskal–Wallis test; Fig. 1k). No significant difference in MUA latency to peak was found between baseline, 0–2 h, and 3–5 h stimulation trials (*H* = 0.04,* p* = 0.84, Kruskal–Wallis test; Fig. 1l).

**Figure 2:**
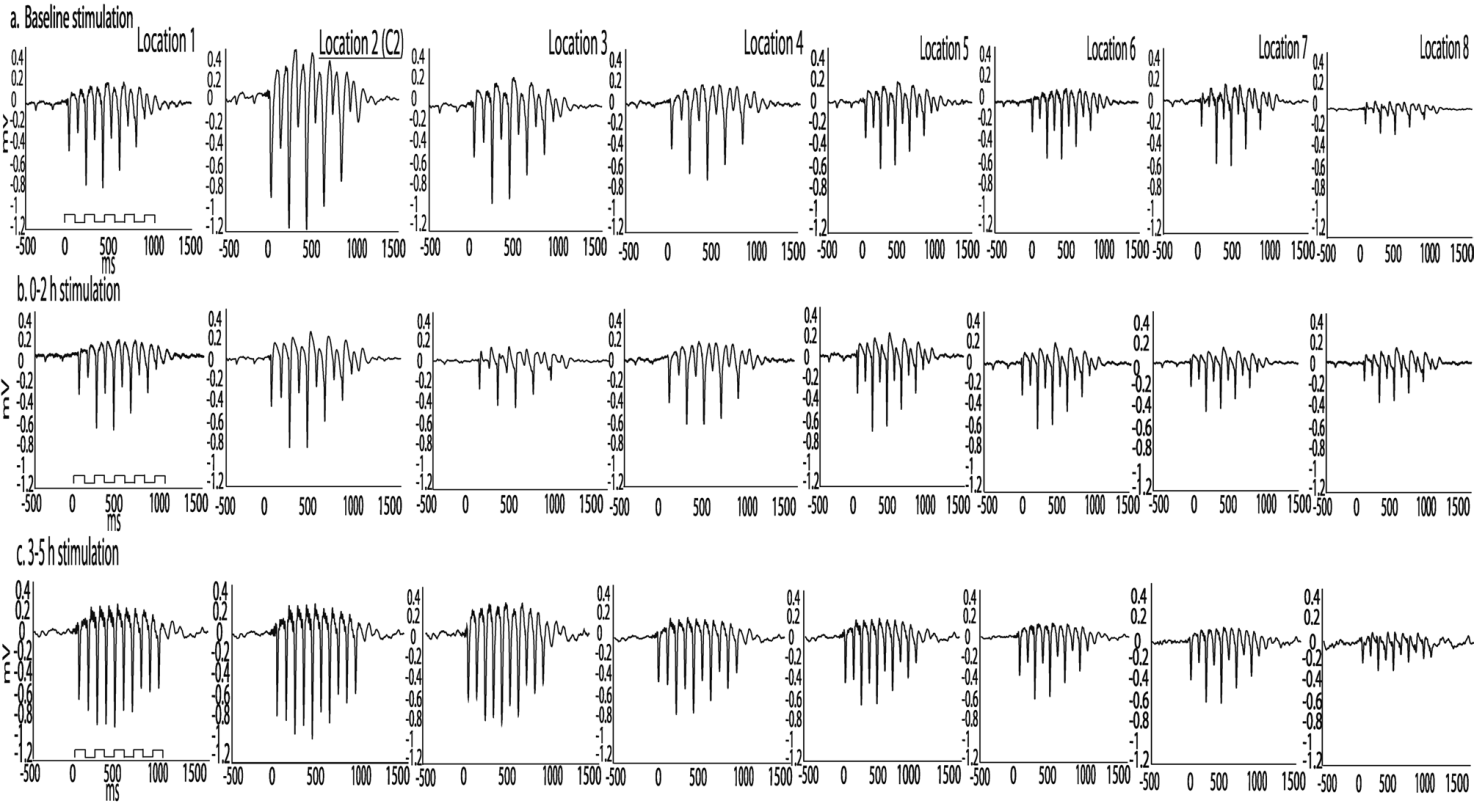
Evoked LFP spread following whisker C2 stimulation was noticeable across all electrodes regardless of when sensory stimulation was delivered. Representative LFP horizontal spread during (**a**) baseline, + 0 h (**b**) and, + 3 h stimulation (**c**) in layer 4 recordings. The step function (left column) represents the 5 pulse 5 Hz train of stimulation delivered for each trial. Evoked LFP averaged across trials for a representative animal are shown for each of 8 horizontal, cortical recording locations. (**a**) and (**b**) represent evoked LFP from different animals each of which underwent 2 h of sensory stimulation delivered at distinct post-pMCAO time points. Evoked LFP decays over distance from peak recording location [underlined location 2 (C2)] but is still present at recording location 8 (3.9 mm from C2 functional representation).

Additionally, comparable to previous findings^14,17^ in the healthy cortex, single whisker stimulation continued to evoke a large LFP activation spread after ischemic onset regardless of when stimulation occurred post pMCAo (Fig. 2). In cortical layer 4 recordings, LFP negative peak magnitude decayed over distance from the peak recording location but extended to the furthest recording location 3.9 mm away from the peak recording location (% of LFP negative peak magnitude 3.9 mm away from peak recording location (100%), at baseline stimulation 19.79% ± 2.84, at 0–2 h stimulation 17.36% ± 2.32, and at 3–5 h stimulation 18.46% ± 2.22). LFP negative peak magnitude decay from peak recording location was not significantly different from baseline regardless of when stimulation was delivered after pMCAo (*H* = 0.51, *p* = 0.78, Kruskal–Wallis test). Consistent with the decay of LFP magnitude over distance from the peak recording location, the latency to LFP negative peak magnitude increased at recording locations further from the peak in all stimulation conditions (latency to negative peak magnitude in furthest electrode, at baseline stimulation 22 ms ± 2.14, at 0–2 h stimulation 22 ± 1.72, and at 3–5 h stimulation 23.16 ± 0.77). No significant difference in latency to negative peak magnitude was observed between baseline, 0–2 h, and 3–5 h stimulation (*H* = 0.85, *p* = 0.32, Kruskal–Wallis test). In 3–5 h stimulation, the LFP positive peak magnitude continued to be distinctly robust and its spread significantly increased from baseline stimulation at the furthest electrode away from the peak recording location (*H* = 7.02, *p* = 0.03, Kruskal–Wallis test, % of LFP positive peak magnitude 3.9 mm away from peak recording location (100%), at baseline stimulation 35.04% ± 2.71, at 0–2 h stimulation 40.70% ± 1.86, and at 3–5 h stimulation 61.92% ± 1.74). A single whisker deflection also evoked large-scale LFP spread in cortical layers 2–3, 5A, and 5B both before and after pMCAo (Supplemental Figure 1).

### Spatiotemporal synchrony of spontaneous LFP increased within minutes after pMCAo and remained high during the acute post pMCAo period

**Figure 3:**
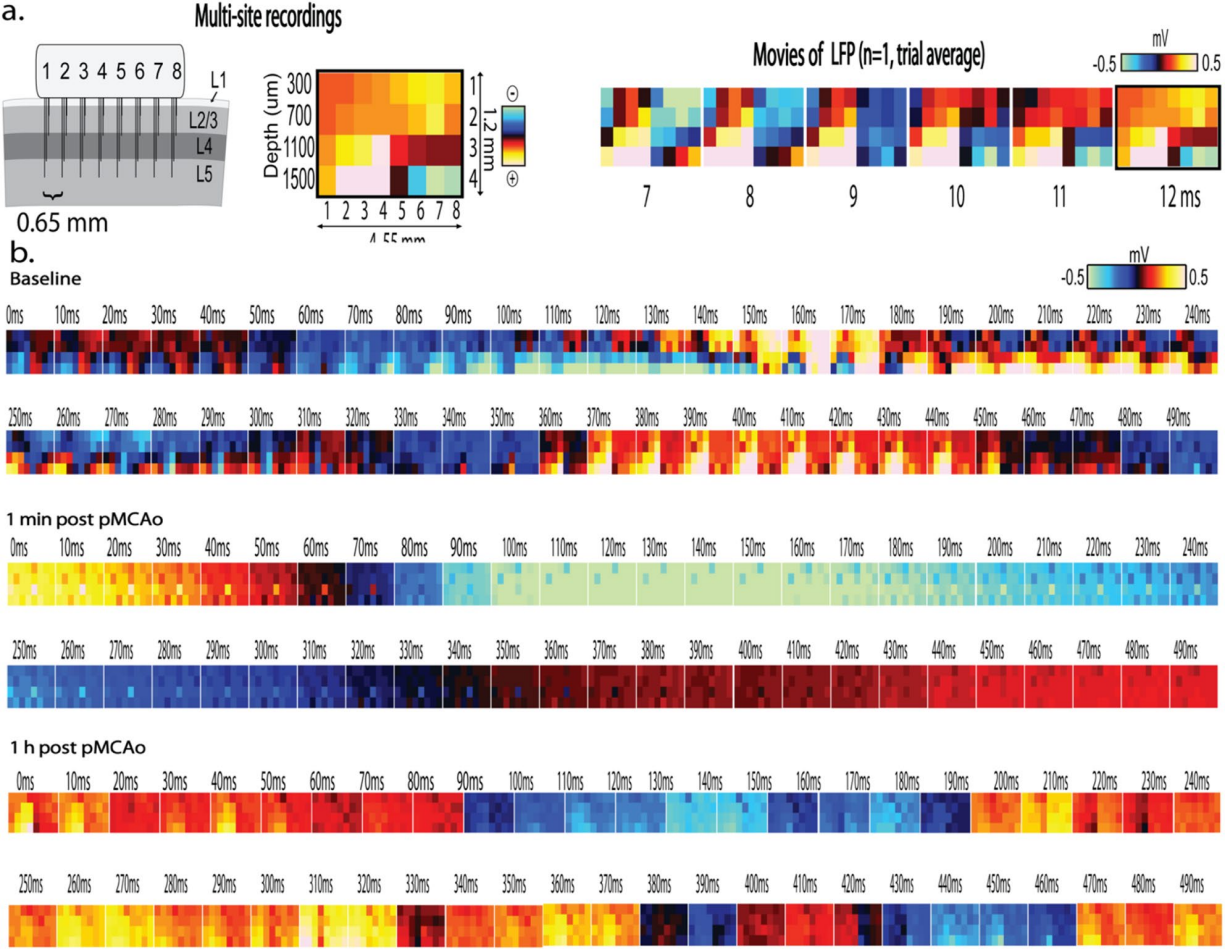
Visualizing 1 ms frames of LFP activity over time reveals spatial and temporal activity patterns after ischemic onset. Robust evoked LFP spread is observed across horizontal locations and cortical depths (Jacobs et al., 2015). (**a**) Multi-site electrode array recordings sample from MCA ischemic territory as shown in Fig. 1. The left panel illustrates the approximate recording locations represented in each frame of activity (middle and right panels). Each microelectrode is represented by a pixel in each frame, and the color of each pixel is indicative of the LFP amplitude at that point in time. Series of 1 ms frames of trial averaged LFP form movies (right panel). (**b**) Representative frames of spontaneous LFP are depicted at fine temporal resolution (10 ms bins) before, 1 min after pMCAo, and 1 h after pMCAo. Complexity of spatiotemporal patterning of activity is abolished after pMCAo, and LFP amplitude across all recording locations and layers sampled demonstrate relative uniformity.

**Figure 4:**
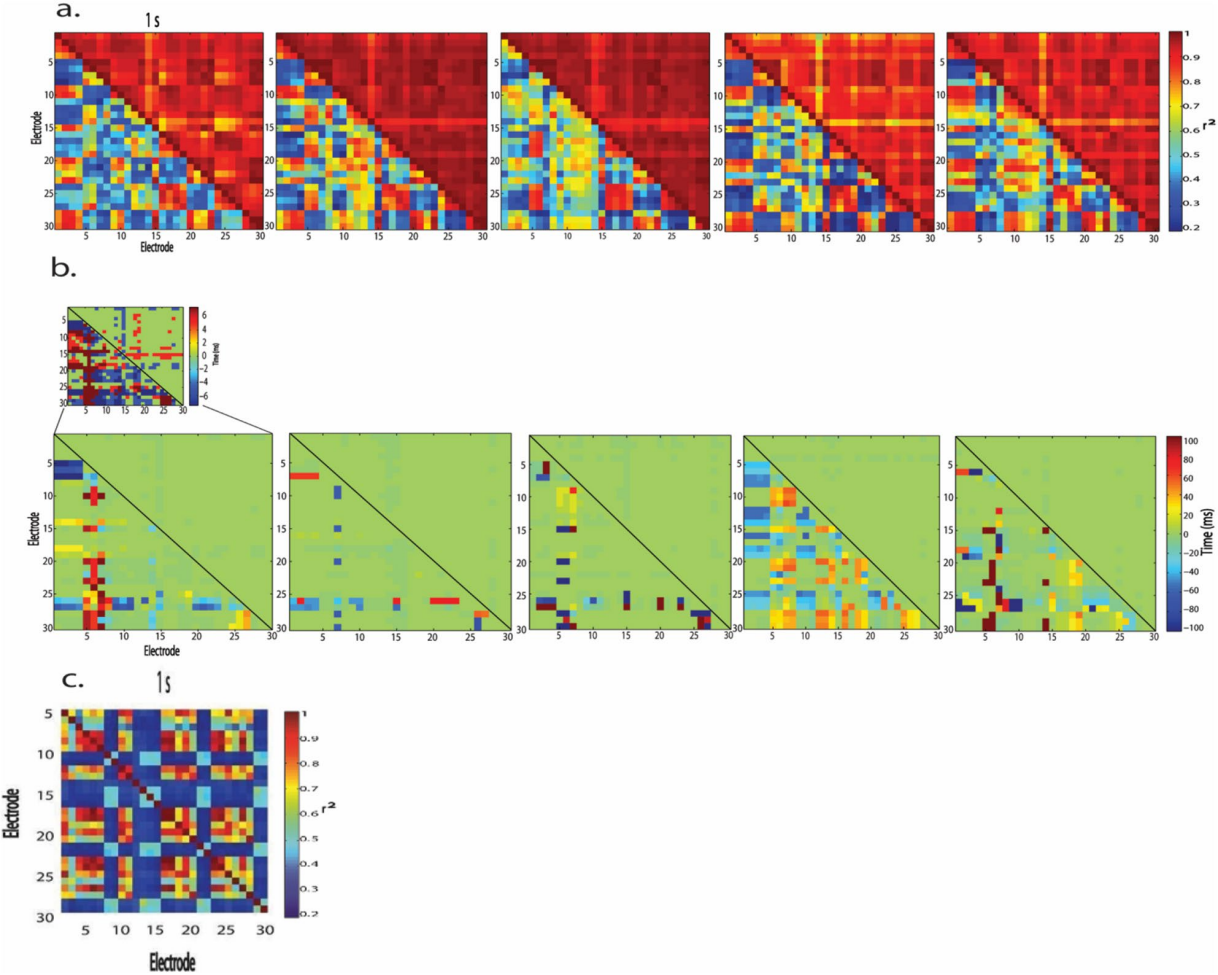
LFP spatial synchrony increases and corresponding time lags decrease directly after ischemic onset. (**a**) Representative LFP cross-correlograms demonstrate the overall increase in cross-correlation coefficients in 5 consecutive 1 s samples during baseline (lower triangular matrix) and within 1 min after pMCAo (upper triangular matrix) in the same animal. Cross-correlograms depict cross-correlation coefficients between each electrode and all other electrodes within the array. Cross-correlation coefficients values are increased post-pMCAo regardless of recording location. (**b**) Time lags corresponding to each r^2^ value in Fig. 1a (baseline in lower triangular matrix and post pMCAo in the upper triangular matrix) show that maximal correlations occur with reduced time lag after ischemic onset. Note, the color scale of time is different between the top and bottom panels of b. The bottom panel in b represents the possible range of time lags whereas the top panel in b represents time lags zoomed in, demonstrating variability in short time lags after pMCAo. (**c**) A representative surgical control cross-correlogram of LFP is depicted from 1 s before (lower left) and after (upper right) baseline in a surgical sham animal demonstrate a range of cross-correlation coefficients between electrode pairs in a healthy cortex. Cross-correlograms illustrate cross-correlation coefficients of each electrode in the electrode array. In surgical sham animals, low and high cross-correlation coefficients were observed throughout recordings. Electrodes with consistent noise were excluded from this representative sample and all analyses.

**Figure 5:**
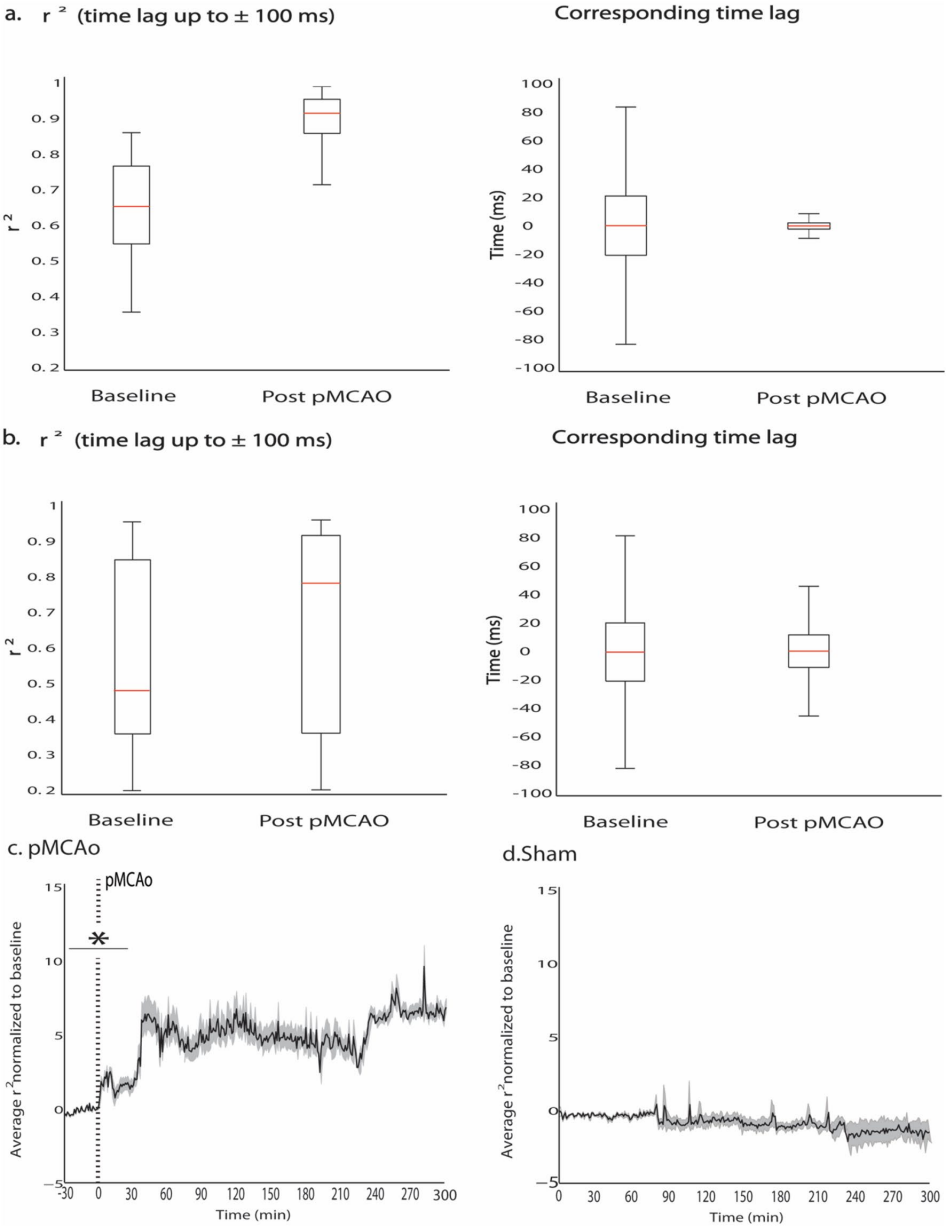
Cross-correlations were used to quantify the increase in spatiotemporal synchrony of spontaneous LFP occurring directly after pMCAo. (**a**) Box and whisker plots of the range of r^2^ values and corresponding time lags in the 10 min before and 10 min directly after pMCAo in the representative case shown in Fig. 4. (**b**) Box and whisker plots of the range of r^2^ values and corresponding time lags from all pMCAo animals (*n* = 7) 10 min before and 10 min directly after pMCAo. The larger range of r^2^ values represented in b as compared to a is likely related to between animal differences in spatiotemporal synchrony onset after pMCAo. (**c**) The average cross-correlation coefficient (baseline normalized) measure increased significantly after pMCAo and was persistently high throughout the acute post pMCAo period (group statistic, *n* = 7, 95% CI = 5.20, 5.47, Bootstrapped t-statistic comparison to null). (**d**) The average cross-correlation coefficient (baseline normalized) measure is consistent throughout 5 h of recordings in surgical sham animals (group statistic, *n* = 4, 95% CI = *− *0.03, 0.02, Bootstrapped t-statistic comparison to null). Both (**c**) and (**d**) represent the average cross-coefficient measure in the solid black line and the standard error between animals in gray shading.

In animals with no sensory stimulation delivered after pMCAo (*n* = 7), spontaneous LFP became highly synchronous across different recording locations directly after pMCAo (Figs. 3 and 4). To quantify the synchrony observed in post pMCAo spontaneous LFP, we cross-correlated each electrode with all others in the electrode array for each 1 s LFP trace before and after pMCAo while allowing for a time lag of up to 100 ms. Each representative cross-correlogram depicted in Fig. 4a was derived from consecutive, non-overlapping 1 s LFP traces. Corresponding time lags from each of these consecutive cross-correlograms are shown in Fig. 4b in which the color bar indicates either the full range of possible time lags (± 100 ms) (Fig. 4b bottom panel) or a zoomed in range of time lags (± 6 ms) (Fig. 4b top panel). The lower triangular matrix in each of Fig. 4a’s cross-correlograms illustrates the extensive range of r^2^ values resulting from comparing baseline electrode pairs. Conversely, the upper triangular matrix of each of Fig. 4a’s cross-correlograms shows that post pMCAo electrode pairs were highly correlated regardless of the spatial locations compared. The corresponding time lags represented in Fig. 4a depict the leading or lagging electrode in each pair and the associated time lag for every (1 s) cross-correlated. Within 1 min after occlusion, the time lags decreased in range (representative case, post pMCAo time IQR decreases 64.3% from baseline, Fig. 5a right; group statistics, pMCAo, *n* = 7, post pMCAo time IQR decreases 45% from baseline, Fig. 5b right) while r^2^ values increased (Figs. 4a and 5a,b, left).

The spatiotemporal synchrony established after pMCAo was further assessed by repeating all cross-correlations throughout the acute post pMCAo period. Considering the similarity of r^2^ values between spatial locations post pMCAo, r^2^ values across the recording array were averaged for each 1 s LFP trace for each animal. To quantify post pMCAo differences from baseline across animals, each 1 s average r^2^ value was normalized to the z-score of the total baseline. Figure 5c,d demonstrates that the significant increase in normalized averaged r^2^ after pMCAo persists throughout the acute post pMCAo period (group statistics, pMCAo, *n* = 7, 95% CI = 5.20, 5.47, Bootstrapped t-statistic comparison to null) but no significant change is observed in the same measure in surgical sham animals (group statistics, surgical sham, *n* = 4, 95% CI = *− *0.03, 0.02, Bootstrapped t-statistic comparison to null). Figure 4c is representative of the varied range of cross-correlations in comparable time points (1 s) before and after baseline in a surgical sham animal. The five consecutive seconds represented illustrate slight cross-correlation increases and decreases over seconds, but cross-correlation changes rarely occur in tandem with many other electrodes and are not increased after baseline.

### Power increased in low multi-frequency bursts during acute post pMCAo period

**Figure 6:**
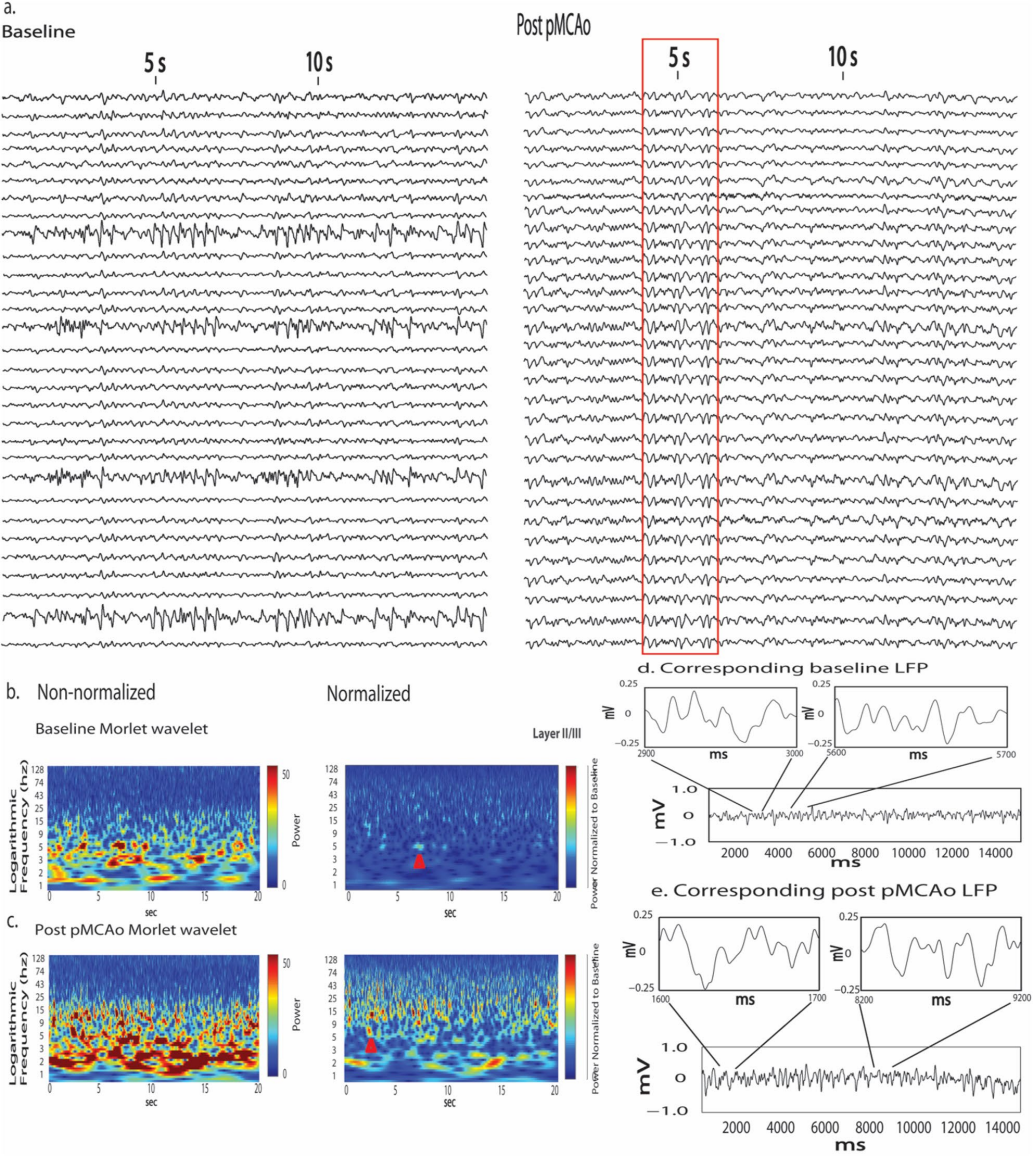
Temporally discrete increases in power (multi-frequency power bursts) are more frequent and are consistently above baseline post pMCAo. (**a**) Representative LFP traces from directly before and within 1 min after pMCAo demonstrate increased similarity between electrodes in spatiotemporal neuronal activity patterns after ischemic onset. As illustrated by the red box, LFP recordings across all electrodes were noticeably spatial and temporally similar post pMCAo at timepoints when multi-frequency power bursts were also observed (**a**, red box). (**b–e**) Representative power spectrum across logarithmically scaled frequencies of the same LFP time series shown for the first electrode in a. Left panels of (**b**) and (**c**) represent the power spectrum in a Layer 2/3 recording directly before (**b**) and within one minute after (**c**) pMCAo (electrode 1 in **a**). Right panels of (**b**) and (**c**) show baseline **(b)** and post pMCAo (**c**) samples after normalization to the z-score of the total baseline. (**d**,**e**) Isolated (above) traces depict time points identified within a multi-frequency power burst and shown by the red arrow in (**b**) and (**c**) respectively.

**Figure 7:**
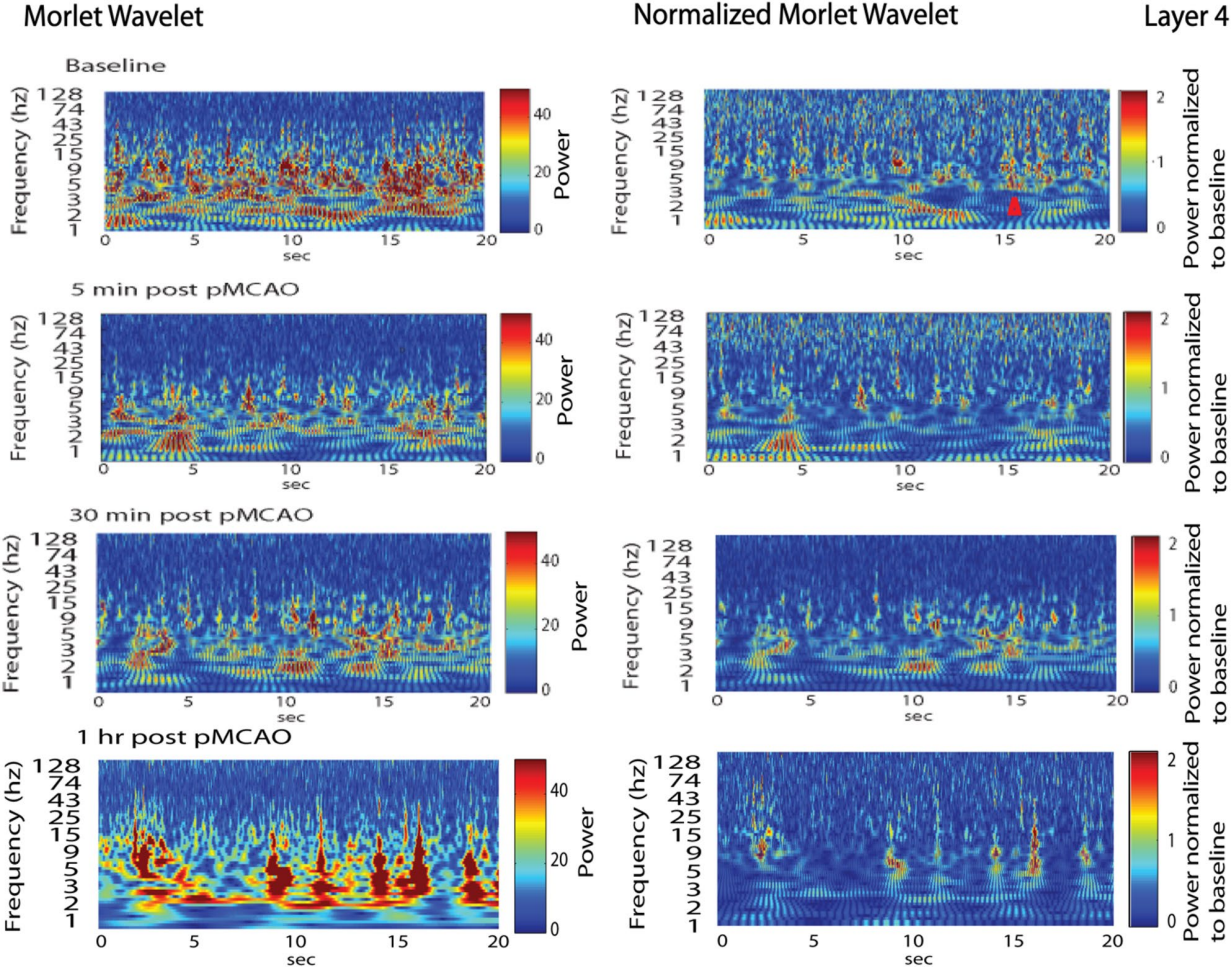
Increased multi-frequency power bursts after ischemic onset continues to be elevated throughout acute ischemic period as shown in a representative Layer 4 electrode 5 min, 30 min, and 1 h post pMCAo. A multi-frequency power burst is highlighted in the red arrow. Power bursts are observed across many frequencies but most notable at lower frequencies. Power bursts continue to be prominent relative to baseline but withstand normalization more at some time post pMCAo timepoints than others.

Morlet wavelet analysis was applied to the LFP during baseline and the acute post pMCAo period to determine whether oscillatory dynamics underlie the increased post pMCAo cross-correlations. Temporally discrete bursts in frequencies’ power were observed across electrodes after pMCAo more frequently than during baseline (Figs. 6, 7). When multi-frequency power bursts were observed, LFP exhibited noticeably similar spatial and temporal activity patterning (Fig. 6a, red box) consistent with Fig. 3. Neuronal activity during power bursts were not epileptiform as is noticeable by the LFP represented in Fig. 6e. Power bursts occurred for approximately 1 or more seconds and were more prominent at lower frequencies, including but not limited to delta band frequencies (Figs. 6c, 7). The presence of multi-frequency power bursts minutes to an hour after pMCAo indicates their persistence during the acute post pMCAo period. Additionally, lower bursts withstood normalization at acute post pMCAo timepoints (Fig. 7).

### Isolated power bursts underlie acute post pMCAo spatiotemporal synchrony

**Figure 8:**
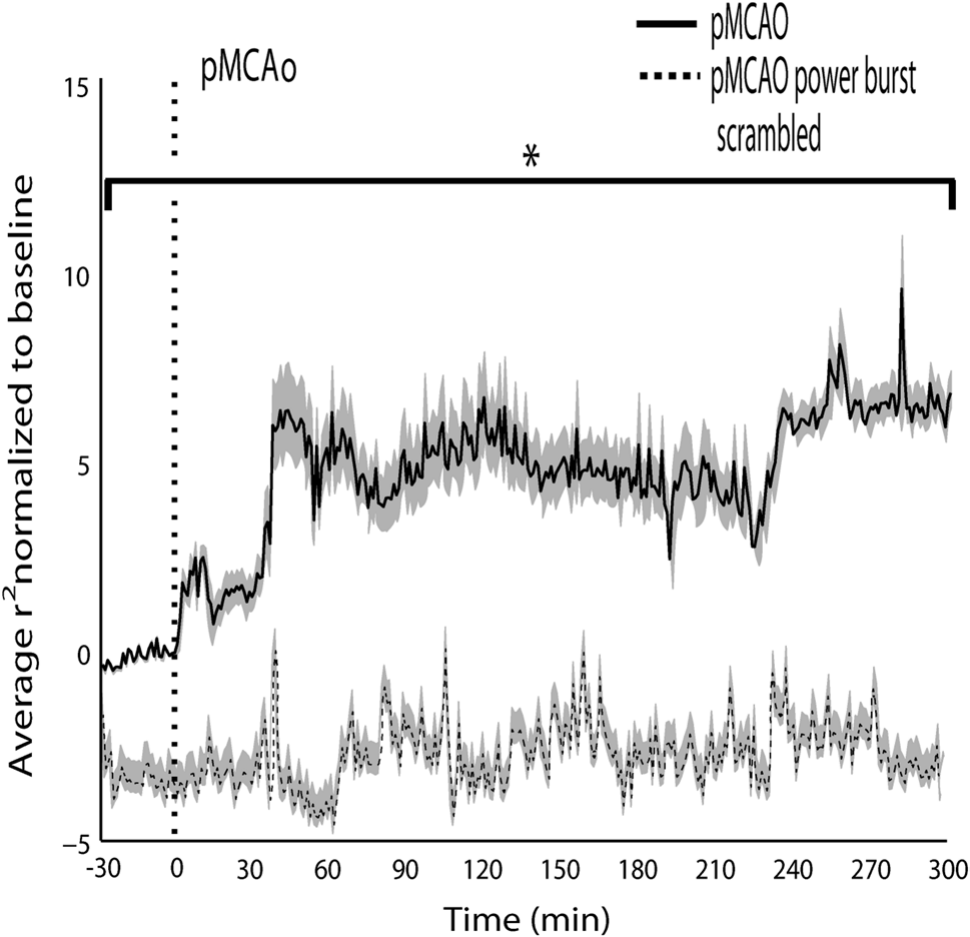
Discrete power burst time points in LFP underlie emergent post pMCAo spatiotemporal synchrony. The solid black line (top, see Fig. 5c) illustrates the average cross-correlation coefficient (baseline normalized) measure that increased directly after pMCAo and was persistently high throughout the acute post pMCAo period (group statistics, pMCAo, *n* = 7, 95% CI = 5.20, 5.47, Bootstrapped t-statistic comparison to null). The dashed black line (bottom) indicates the resulting average cross-correlation coefficient measure when time points involved in power bursts were scrambled (group statistics, pMCAo scrambled, *n* = 7, 95% CI = *− *0.4, 0.89, Bootstrapped t-statistic comparison to null), demonstrating that bursts are necessary for elevated post pMCAo spatiotemporal synchrony. Gray shadings for the black line and shaded line indicate the standard error.

Temporally discrete increases in power were isolated in LFP recordings and identified as a power burst when power exceeded the RMS power threshold of each frequency for 100 or more consecutive ms. LFP was then randomly scrambled during each power burst and concatenated with non-bursting LFP time points for each electrode for cross-correlations to be rerun on the modified LFP. When averaged across recording locations and repeated over time before and after pMCAo in a way comparable to Fig. 5c, average cross-correlation coefficients no longer increased after pMCAo and remained comparable to baseline throughout the acute post pMCAo period (Fig. 8) (group statistics, pMCAo scrambled, *n* = 7, 95% CI = *− *0.4, 0.89, Bootstrapped t-statistic comparison to null). Figure 8 displays the average cross-correlation coefficients across animals from baseline to post pMCAo without power burst scrambling and with power burst scrambling to highlight the causal effect of power bursts in acute post pMCAo spatiotemporal synchrony.

### Significant cortical cell death by 24 h post pMCAo

24 h after ischemic onset significant infarct damage was observed in pMCAo animals as compared to animals with surgical sham preparations (group statistics, pMCAo, *n* = 7, 19.47 mm^3^ ± 2.18, surgical sham, 3.6 mm^3^ ± 1.13; *p* = 0.0095, Wilcoxon signed rank test, Supplemental Figure 2). The percent of infarct volume to total cortical volume was also significantly higher in pMCAo animals (group statistics, pMCAo, *n* = 7, 13.08% ± 0.66, surgical sham, 1.4% ± 0.44; *p* = 0.0061, Wilcoxon signed rank test). Supplemental Figure 3 shows that electrodes sampled neuronal activity within the MCA territory where stroke damage occurred.

## Discussion

Current research is the first study to generate a continuous spatiotemporal profile of neuronal activity directly after ischemic onset and thoroughly examine the evolution of spontaneous and evoked neuronal activity in the minutes and hours after distal MCA occlusion. The slow progression of evoked response changes after pMCAo suggests that stroke damage develops several hours after ischemic onset in this model potentially due to collateral flow capable of maintaining cortical viability after the ischemic event^9,16,21^. In contrast, strong, large scale spatiotemporal synchronization of spontaneous neuronal activity is modulated within minutes of ischemic onset.

The rate of neuronal depression in the ischemic cortex is known to be highly correlated with ischemic severity^22–24^. Indicative of the moderate ischemic insult resulting from pMCAo, Lay et al., 2011 demonstrated in this model a 74% ± 4 decrease in blood flow directly after ischemic onset and a comparable range of infarct volumes. A recent quantitative measurement in the same rat model using Doppler optical coherence tomography (Doct) demonstrated that indeed flow velocity following pMCAo dropped to 11.4% of baseline and flux dropped to 8.2% of baseline^21^. Importantly, if blood flow decreases by < 80% of baseline, stroke damage can be reversed but reversibility is time-limited^25^. Robust evoked MUA and LFP during the acute post pMCAo period is unique to this animal model and may explain the reversal of impending stroke damage known to occur in this model when sensory stimulation is provided within the first 2 h after pMCAo^9^.

Although it is unclear why particular measures of evoked response properties were increased but not others during 3–5 h stimulation, the tendency for high MUA peak firing rate and the significant increase in LFP positive peak magnitude and evoked spread measures in 3–5 h stimulation indicate that evoked activity continues to be robust even hours after pMCAo. Significantly increased LFP positive peak magnitude from 3 to 5 h post pMCAo does not necessarily indicate either hyperexcitability or silencing as the same subpopulation of co-activated synapses can produce distinct current sources^26^.

Despite relatively intact evoked responses, spontaneous spatiotemporal synchrony was significantly increased throughout acute ischemia following pMCAo whereas spontaneous spatiotemporal synchrony was unchanged after baseline in surgical sham animals. The persistence of heightened spatiotemporal synchrony in acute ischemia is critical to the reliability of the measure as many ischemic pathophysiological indicators rapidly change and are only transiently quantifiable^10^. Abnormal synchrony has also been identified in other neurological disorders and is thought to attribute to associated pathophysiology (e.g., schizophrenia, epilepsy, Alzheimer’s disease, and autism)^27,28^. The temporal coordination of neuronal activity is hypothesized to give rise to large-scale functional network formation and maintenance. Accordingly, when synchrony dynamics are modulated, pathological states emerge that may underlie specific symptoms of neurological disorders (e.g., increased EEG synchrony during and between absence epileptic seizures)^29,30^. Identifying abnormal spatiotemporal synchrony in acute ischemic stroke provides insight into a unique similarity between the large-scale networks of diverse neurological conditions and demonstrates the rapid rate large-scale functional impairments occur after brain insult (i.e., occlusion onset).

Complex Morlet wavelet analysis was utilized to determine the time-varying frequency specific power changes underlying emerging post pMCAo spatiotemporal synchrony. Similar in time course to elevated post pMCAo spatiotemporal synchrony trends, low multi-frequency power bursts became predominant within minutes after pMCAo and remained increased after pMCAo. Similar long bursts have been observed in synchronized spontaneous cortical activity of other animal models of neurological conditions^31^. Post pMCAo power bursts are congruous with non-epileptiform intermittent delta rhythmic activity (IRDA) observed in human ischemic EEG. IRDA is an electrophysiological feature closely associated with a wide variety of lesion and metabolic disturbances^32,33^. Resembling IRDA, multi-frequency LFP power bursts are non-epileptiform and involve but are not exclusive to delta band frequencies. The observation of multi-frequency power bursts potentially validates our animal stroke model by replicating an established clinical biomarker of neurological dysfunction. IRDA, however, may differ from the described multi-frequency power bursts in localization: IRDA is most commonly observed in frontopolar EEG leads but can also be diffuse^32^.

Although neuronal network dynamics are rarely studied in stroke, increased spatiotemporal synchrony after ischemia is consistent with low frequency synchrony recorded in perilesional cortex days after focal ischemic onset^34,35^. The increased spatiotemporal synchrony observed within minutes after pMCAo extend and expand these initial findings by describing the evolution and persistence of spatiotemporal synchrony for the 5 h directly after ischemic onset. Additionally, the current research is distinct in evaluating the scale of spatiotemporal synchrony during ischemia. Our results demonstrate that the synchrony established after ischemic onset but not in surgical sham controls occurs throughout the MCA territory and spans cortical depths. Taken together, our studies indicate that dysfunctional spatiotemporal activity coordination may be a long-lasting signature of ischemia resulting in future stroke damage.

Future recordings will be required to determine the cortical extent of post pMCAo synchrony and whether it is pervasive in other stroke models including awake stroke models. An awake model of mini strokes found that spatiotemporal neuronal deficits extend beyond cerebral blood flow restoration in the days and weeks after ischemic onset^36^. Future studies should examine whether spatiotemporal synchrony extend in the long-term after pMCAo and if these neuronal network dynamics are related to residual blood flow dynamics. Additional studies are also necessary to determine if the cortical extent of post pMCAo synchrony predicts the magnitude of ischemic stroke damage. This relationship could not be determined using our set up because damage due to stroke and damage resulting from electrode placement was indistinguishable.

When LFP at time points involved in power bursting was scrambled, LFP cross-correlation coefficients remained at baseline levels after ischemic onset. Thus, power bursting is a mechanism underlying elevated post pMCAo spatiotemporal synchrony and intermittent oscillatory activity is capable of driving large-scale temporal coordination of ischemic neuronal networks. Like IRDA, high spatiotemporal synchrony may then be a biomarker of ischemia and indicative of stroke damage. The emergence of elevated synchrony before irreversible stroke damage makes spatiotemporal synchrony a particularly attractive ischemic biomarker as it may precede the ability to visualize neural injury using neuroimaging diagnostics (e.g., magnetic resonance imaging).

Although it is possible that the sodium pentobarbital used as an anesthetic in this study affected post pMCAo spatiotemporal synchrony and underlying power bursts, post pMCAo recordings were compared to baseline recordings and surgical sham control animals under the same anesthesia to control for any effects due to anesthesia. As sodium pentobarbital is known to suppress neuronal activity bursting in other rat models of focal cerebral ischemia^37^, the persistence of high spatiotemporal synchrony and power bursts in our anesthetized model suggests that these spatial and temporal neuronal activity patterns would continue to be robust and may even be heightened in awake preparations.

As previously reported in this ischemic animal model, sensory stimulation is capable of protecting the cortex from ischemic damage^9,16,21,38–40^. Future studies will investigate whether synchrony and intermittent oscillations are changed in sensory protected animals to further determine whether these electrophysiological features are signatures of impending damage.

If our LFP spontaneous activity measures are translatable to human EEG, identified electrophysiological biomarkers of ischemia could be important for evaluating the functional effects of ischemia rapidly in a clinical setting. Electrophysiological and neuroimaging measures together more accurately predict stroke symptoms than neuroimaging alone^6,7,41^. Indeed, a recent study of suspected stroke cases has demonstrated that delta band power and alpha/delta frequency ratio of EEG recorded in the emergency department using a dense-array (256 lead) system could predict large stroke damage^42^. Other studies^43–48^ have identified potential EEG biomarkers of clinical status or motor impairment, and even to predict recovery in the period after stroke^44,49^. We find that across these studies different frequency bands (typically delta, alpha, and beta), different electrode locations, and different measures (spectral power, coherence, phase synchrony, etc.) have been used to show the potential of EEG in understanding brain injury in stroke^43–50^. These studies are difficult to synthesize because of the heterogeneity of the size and location of strokes in humans and the varying time periods used in experiments (within hours (acute), weeks (subacute) or months (chronic) of the stroke)^50^. Indeed, our own experience shows that useful biomarkers in acute patients at the Emergency Room^46^ are quite different from subacute and chronic patients^50^.

Since EEG is generated by the same mechanisms as cortical LFP, we suggest that our rat model could be relevant for understanding EEG in stroke in humans and assist in the identification of meaningful biomarkers in the acute phase where intervention is possible. Our results and these clinical EEG findings suggest future potential electrophysiological biomarkers that can be measured immediately after ischemic onset which may be critical to advancing characterizations of ischemic cortical function and improving stroke impairment predictions.

## Supplementary information

Supplementary Figures.

## Data Availability

The datasets generated during and/or analyzed during the current study are available from the corresponding author on reasonable request.
