# Author Correction: Rapid development of strong, persistent, spatiotemporally extensive cortical synchrony and underlying oscillations following acute MCA focal ischemia

**DOI:** 10.1038/s41598-021-86715-z

**Published:** 2021-03-24

**Authors:** Ellen G. Wann, Anirudh Wodeyar, Ramesh Srinivasan, Ron D. Frostig

**Affiliations:** 1 Department of Neurobiology and Behavior, University of California, Irvine, CA USA; 2 Center for the Neurobiology of Learning and Memory, University of California, Irvine, CA USA; 3 Department of Cognitive Science, University of California, Irvine, CA USA; 4 Department of Statistics, University of California, Irvine, CA USA; 5 Department of Biomedical Engineering, University of California, Irvine, CA USA

Correction to: *Scientific Reports* 10.1038/s41598-020-78179-4, published online 08 December 2020

The Supplementary Information published with this Article contains an error where redundant Supplementary Figure legends are present.

The correct Supplementary Information file is provided below.

## Supplementary Information

Supplementary Information.

