# Supplementary material for: Rapid development of strong, persistent, spatiotemporally extensive cortical synchrony and underlying oscillations following acute MCA focal ischemia
Source: Sci Rep. 2020 Dec 8;10:21441. doi: 10.1038/s41598-020-78179-4 (PMC7722868; doi:10.1038/s41598-020-78179-4)

Rapid development of strong, persistent, spatiotemporally extensive cortical synchrony and underlying oscillations following acute MCA focal ischemia

Authors: Ellen G Wann^1,2^, Anirudh Wodeyar^3,4^, Ramesh Srinivasan^3,4^, Ron D Frostig*^1,2,5^

Author affiliations:

^1^ Department of Neurobiology and Behavior, University of California, Irvine, CA, USA

^2^ Center for the Neurobiology of Learning and Memory, University of California, Irvine, CA, USA

^3^ Department of Cognitive Science, University of California, Irvine, CA, USA

^4^ Department of Statistics, University of California, Irvine, CA, USA

^5^ Department of Biomedical Engineering, University of California, Irvine, CA, USA

**Supplemental Figure 1.** Representative frames of evoked LFP before and after pMCAo in a 0 to 2 h stimulation animal **(A)** and a 3 to 5 h stimulation animal **(B)**. Evoked activation spread for the trial mean of the animal represented can be seen in 1 ms LFP movie frames after the first pulse of each 5 pulse train for a total of 25 ms. The spread of LFP activity remains large and radiates symmetrically whether stimulation is delivered prior to ischemic onset, 0 to 2 h after pMCAo, or 3 to 5 h after pMCAo.

**Supplemental Figure 2.** Histological assessment of infarct volume 24 h after pMCAo demonstrates significant cortical infarct in pMCAo animals compared to surgical shams controls (**p<0.05*). Damage observed in surgical shams is likely attributed to MCA craniotomy or electrode placement.

**Supplemental Figure 3.** Representative flattened cortical section shows horizontal electrode locations relative to the infarcted (bounded) region.

**Supplemental Figure 1:** Spatial and temporal evoked LFP before and after pMCAo

**Supplemental Figure 2:** Significant infarct volume after pMCAo

**Supplemental Figure 2:** Significant infarct volume after pMCAo

**Supplementary Figure 1**


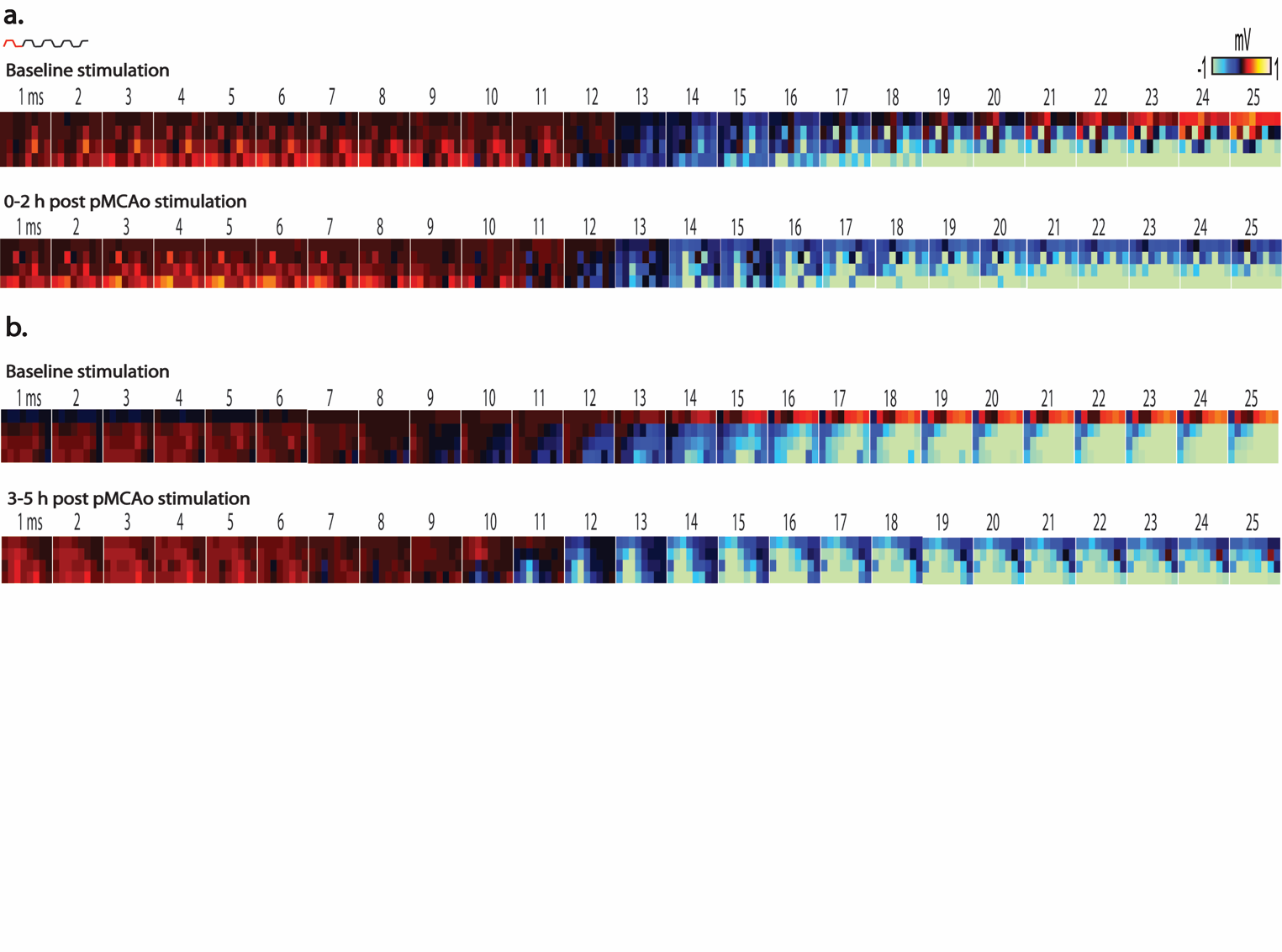


**Supplemental Figure 2:** Significant infarct volume after pMCAo

**Supplemental Figure 2:** Significant infarct volume after pMCAo


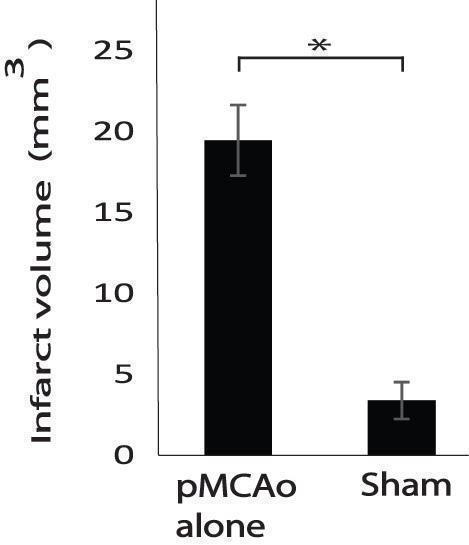


**Supplemental Figure 3**: Electrode array placement relative to infarct location


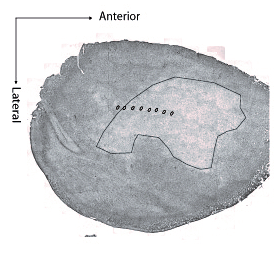

Supplement: Supplementary file 1 — Supplementary Figures. [file 41598_2020_78179_MOESM1_ESM.docx]
